# Delayed Fluorodeoxyglucose Positron Emission Tomography Imaging in the Differentiation of Tumor Recurrence and Radiation Necrosis in Pediatric Central Nervous System Tumors: Case Report and Review of the Literature

**DOI:** 10.7759/cureus.3364

**Published:** 2018-09-26

**Authors:** Elizabeth L Wadhwa, Benjamin L Franc, Mariam Aboian, John Y Kim, Miguel Pampaloni, Theodore Nicolaides

**Affiliations:** 1 Pediatrics, University of California, San Francisco, USA; 2 Radiology, University of California, San Francisco, USA; 3 Neurosurgery, Kaiser Permanente, Oakland, USA; 4 Pediatrics, New York University Langone Medical Center, New York, USA

**Keywords:** delayed fdg pet imaging, pediatric central nervous system tumors

## Abstract

Malignant central nervous system (CNS) tumors are often treated with radiation therapy, after which clinical and radiographic sequelae can lead to difficulties differentiating tumor recurrence from treatment effect. Magnetic resonance imaging (MRI) is often unable to distinguish between these two entities. The improved ability to delineate concerning foci could lead to earlier tumor detection with quicker access to new therapies and/or clinical trials; conversely, it could alleviate patient concerns in the case of radiation necrosis as the etiology. The utility of positron emission tomography with computed tomography (PET/CT) imaging with fluorodeoxyglucose (FDG) has been explored in CNS tumors in the past, as this imaging modality is widely used in oncologic practices. As there are concerns with false positive imaging in the case of cells with a high metabolic uptake due to causes other than malignancy (i.e. infection, inflammation), delayed FDG PET imaging has been proposed as a mechanism to reduce this confusion. Delayed FDG PET imaging has been explored in several adult and pediatric malignancies, including adult CNS tumors, though there are no current publications applying its use in pediatric CNS tumors. We present two cases of pediatric CNS tumors, where delayed FDG PET imaging helped in the early diagnosis of a recurrence through a distinguishing tumor from the treatment effect.

## Introduction

Many central nervous system (CNS) tumors are treated with radiation therapy. Treatment-induced radiation necrosis can have clinical symptoms and radiographic findings very similar to brain tumor recurrence. Radiation injury has been estimated to occur in 5% to 37% of cases and can be difficult to differentiate from residual or recurrent malignancy by magnetic resonance imaging (MRI). Positron emission tomography (PET) has been used to differentiate radiation injury from recurrent or residual malignancy on the basis of differences in glucose uptake [1]. Verma et al. summarized the ability of PET imaging to differentiate between tumor and therapy effect. They summarized prior reports, finding that FDG PET has been reported to be capable of distinguishing treatment necrosis from tumor recurrence with sensitivity and specificity in the ranges of 65%-81% and 40%-94%, respectively. They noted the same limitations of this imaging modality that were previously recognized, including variable FDG uptake in the normal cortex and the propensity for low-grade tumors to appear metabolically similar to normal brain tissue [2]. Chao and colleagues reported that MRI co-registration appears to improve the sensitivity of FDG PET from 65% to 86%, making it a useful modality to distinguish between radiation necrosis and recurrent brain metastasis [3]. Zhuang et al. looked at the appearance of radiation necrosis on imaging. In a cohort of patients previously treated with bevacizumab, MRI or spectroscopy was performed first, followed by PET/computed tomography (CT) as needed for ambivalent diagnoses. They found that PET showed a lower rate of glucose uptake and a lower metabolic rate in radiation brain necrosis lesions as compared with normal brain tissue [4]. Kumar et al. compared the accuracy of MR perfusion and FDG PET in differentiating tumor progression from a non-neoplastic contrast-enhancing tissue. Their retrospective review included 23 cases of primary brain tumors and five metastatic lesions with enhancing lesions seen on post-treatment MRI. They found a recurrent tumor to demonstrate a significantly higher cerebral blood volume (CBV) in MR perfusion and standardized uptake value (SUV) in FDG-PET than non-neoplastic contrast-enhancing tissue, though MR perfusion had greater accuracy [5].

PET and PET/computed tomography (PET/CT) imaging with fluorodeoxyglucose (FDG) are imaging modalities that are used with great frequency for diagnostic and surveillance purposes in current oncology practice. The uptake by cells of FDG is in part regulated by the number of glucose transporters on cells as well as the metabolic rate of the cell. Malignant cells often have a high metabolism and increase the number of glucose transporters, which lend to increased FDG accumulation within them, allowing FDG PET to produce the useful and important images to which oncologists have become accustomed. As with many facets of medicine, there is a downside, which lies in the fact the FDG is also taken up by other cells that have a high metabolic rate. Cells with a high FDG uptake can also include those that are infected or are in an active inflammatory state. In cases where oncology patients have infection or inflammation concurrent with malignancy, which is often the case due to the immunocompromised state or dysregulated inflammatory state or as an effect of treatment, the results of FDG PET imaging can be quite confusing. In order to alleviate these concerns and maintain the utility of this imaging, physicians and scientists have studied the potential of delayed or dual timepoint FDG PET imaging. The thought behind such imaging is that malignant cells will increase FDG over a period of time while infected or inflamed cells will decrease uptake, with the goal of being able to distinguish benign and malignant processes [6]. Delayed FDG PET imaging has been explored in several adult and pediatric malignancies, including adult CNS tumors, though it has not yet been evaluated in pediatric CNS tumors. We present two cases of pediatric CNS tumors, where delayed FDG PET imaging helped in the early diagnosis of recurrence by distinguishing tumor from treatment effect.

## Case presentation

**Figure 1:**
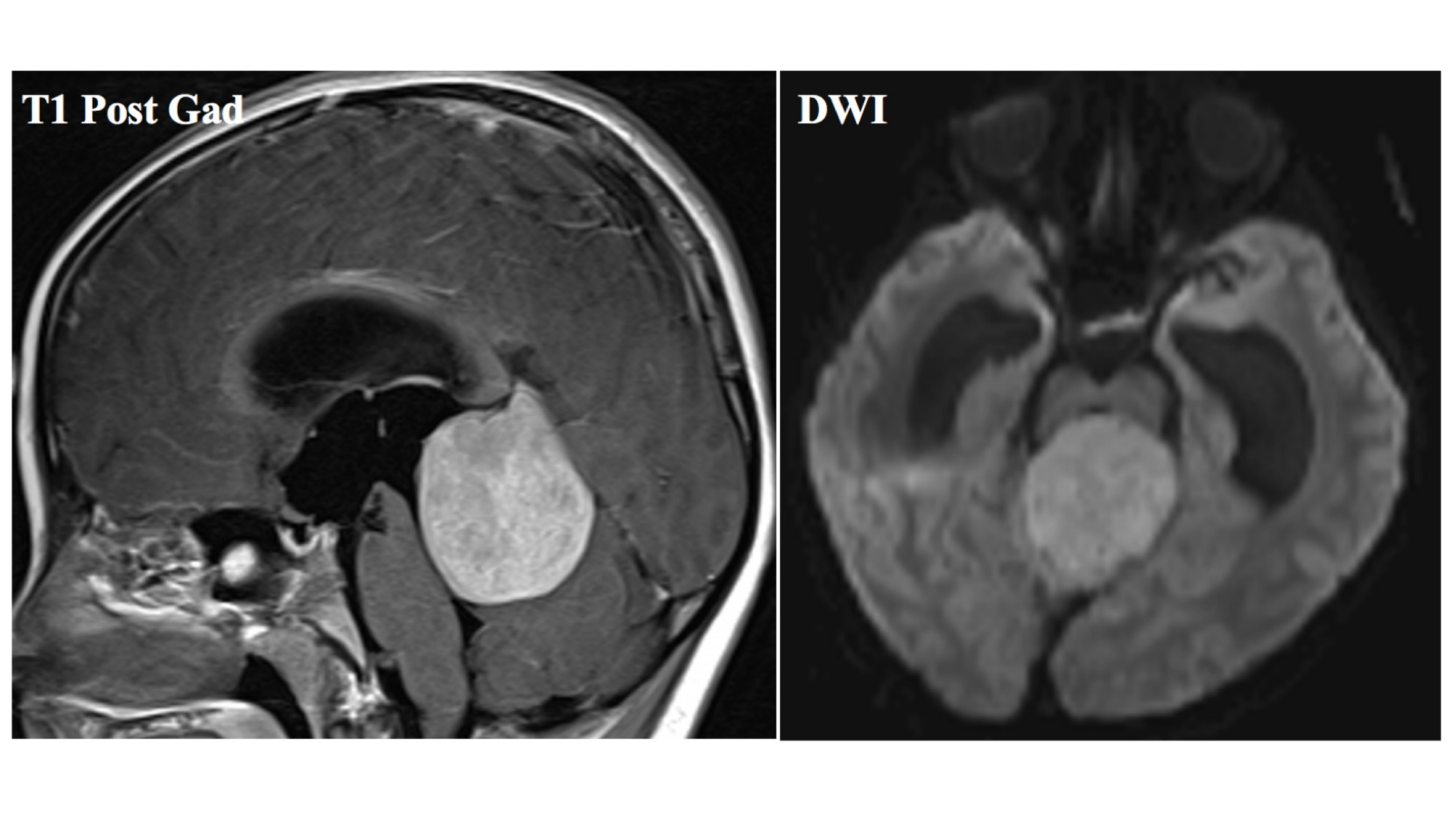
High-grade pineal parenchymal tumor at presentation Demonstrates an avidly enhancing mass involving the pineal gland with mass effect on the cranial portion of the vermis. The mass demonstrates evidence of reduced diffusion on DWI, suggesting increased cellularity within the tumor. DWI: diffusion-weighted imaging

**Figure 2:**
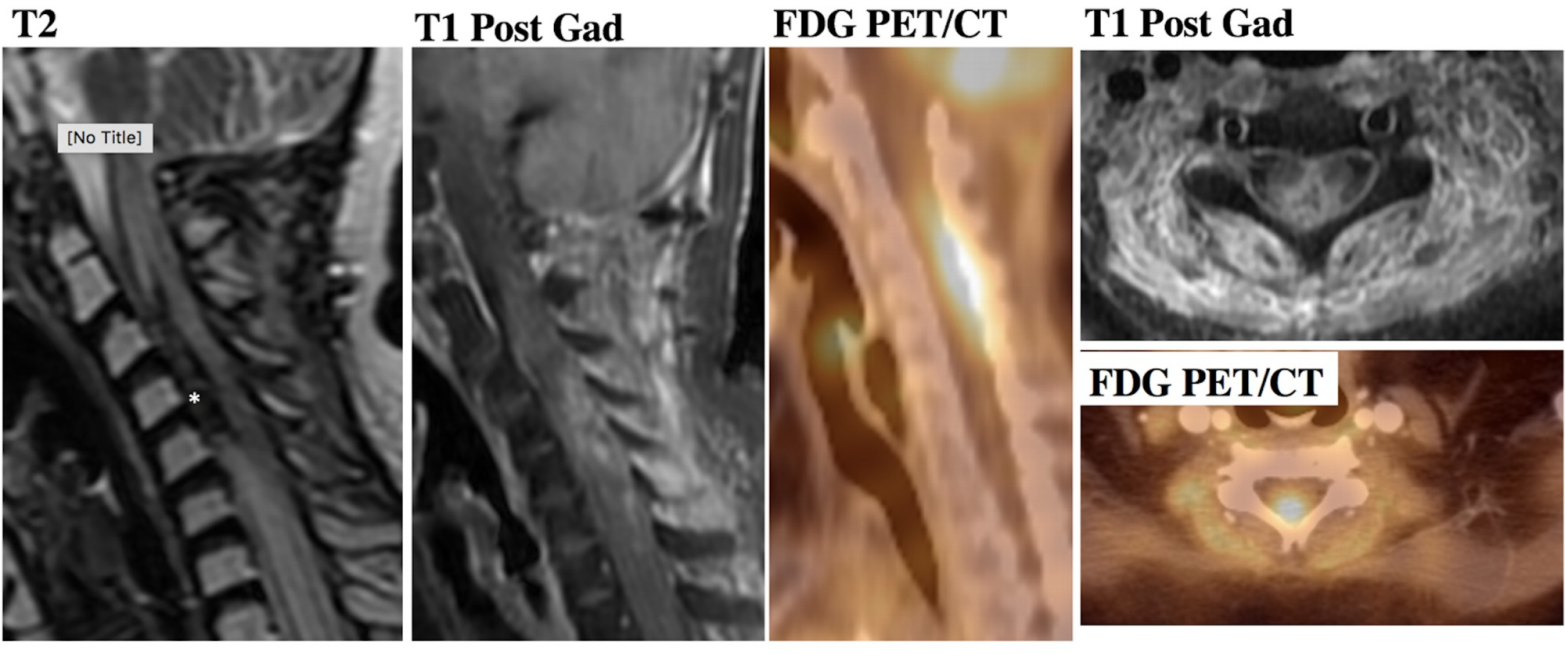
High-grade pineal parenchymal tumor in a patient with disseminated disease to the spine Region of enhancement of the cervical spinal cord spanning from C2 to C7 with evidence of blood products indicated by an asterisk (*). 18F-FDG PET demonstrates avid uptake of the tracer within the enhancing portion of the spinal cord, indicating a recurrent tumor. 18F-FDG PET: 18F-fluorodeoxyglucose positron emission tomography

Patient A was diagnosed with a pineal parenchymal tumor of intermediate differentiation at age seven, after presenting with headache, vomiting, and gait disturbance (Figure 1). Lumbar puncture and spine MRI at the time of diagnosis were negative for disseminated disease. She underwent gross total resection, and though her postoperative course was complicated by severe brainstem dysfunction requiring tracheostomy and gastrostomy tube placement, she had a remarkable recovery with intensive rehabilitation, allowing for tracheostomy decannulation and the removal of her feeding tube. She underwent treatment as per Children’s Oncology Group protocol ACNS0332 (looking at the efficacy of carboplatin administered concomitantly with radiation in high-risk medulloblastoma and primitive neuroectodermal tumors) with 5400 cGy of whole ventricular irradiation and chemotherapy with cisplatin, vincristine, and cyclophosphamide. Ten months after completing therapy, she was noted on routine surveillance imaging to have a 4x7x5 mm enhancing nodule in the ventral spine at C3. Her brain MRI and CSF showed no further sites of disease. At this time, she underwent radiation therapy of her cervical and thoracic spine to the level of T6. She had a near-complete response to induction chemotherapy as per ACNS0334 (Phase III trial looking at intensive induction chemotherapy with methotrexate followed by consolidation with stem cell rescue versus the same therapy without methotrexate). She then completed two cycles of consolidation chemotherapy with carboplatin and thiotepa followed by autologous stem cell rescue. She developed thrombotic microangiopathy as a complication following her second stem cell infusion, so she did not receive her third cycle of high-dose chemotherapy with stem cell rescue as originally planned, though she did require a stem cell boost for persistent reticulocytopenia and thrombocytopenia three months after her second. Three months following her last round of chemotherapy, the patient underwent a disease evaluation with total spine and brain MRI, which revealed a new small enhancing lesion at C4-5 concerning for disease relapse versus radiation-induced change. A short interval follow-up MRI was performed three weeks later, again concerning for radiation effect versus disease recurrence. Delayed PET imaging performed at this time showed linear focal hypermetabolism in the dorsal aspect of the spinal canal extending from C3-C5 corresponding to the previously described area of ill-defined nodular enhancement described on the spine MRI and highly concerning for recurrent malignancy (Figure 2). The sequela of radiation therapy was deemed a less likely consideration, given the hypermetabolism in this region. The spine MRI obtained shortly thereafter clearly showed a tumor. The patient developed progressive neurologic deficits with worsening upper extremity tremors followed by progressing bilateral upper and lower extremity weakness as well as stool incontinence. Her motor exam improved with steroids and was ultimately transitioned to her local institution for palliative radiation and continued care.

**Figure 3:**
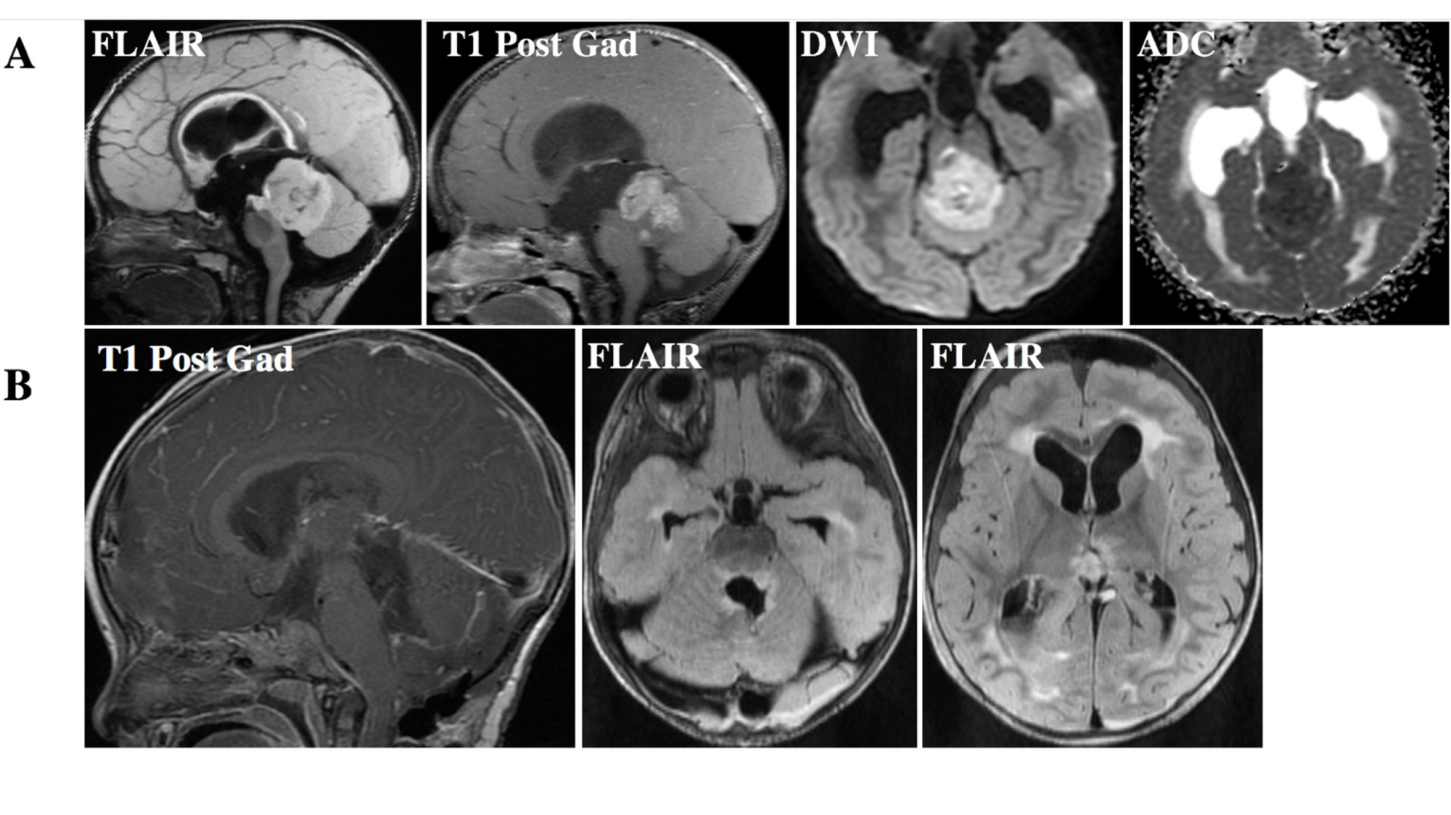
Pineoblastoma with the DICER mutation at presentation and following resection A. FLAIR hyperintense and enhancing lesion involving the pineal gland with inferior displacement of the vermis. The lesion demonstrates prominent reduced diffusion, indicating high cellularity. B. Evidence of gross total resection of the tumor on post-gadolinium imaging. FLAIR imaging demonstrates post-surgical changes within the resection bed and evidence of an intraventricular hemorrhage. FLAIR: fluid attenuated inversion recovery

**Figure 4:**
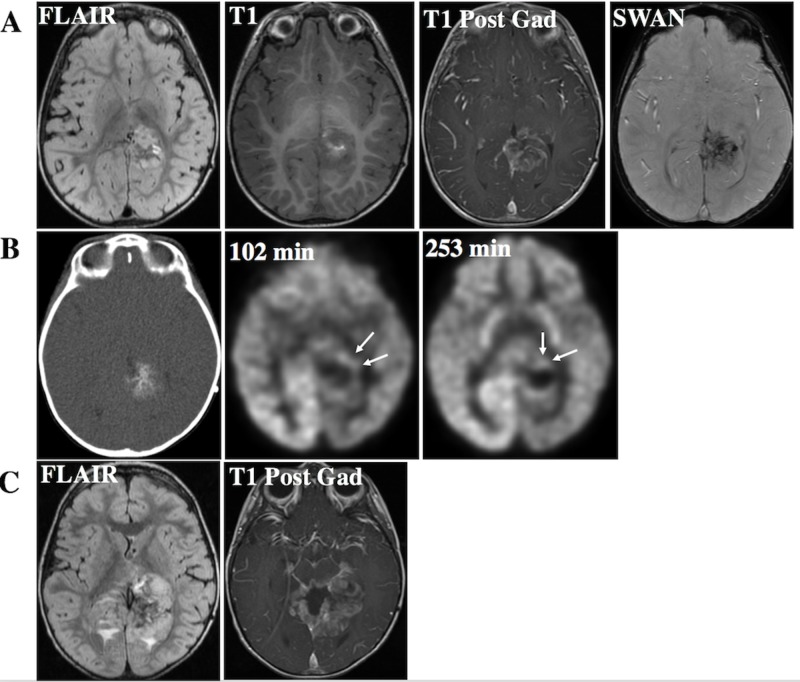
Pineoblastoma with the DICER mutation on follow-up A. FLAIR hyperintense and enhancing lesion within the left posterior cingulate gyrus with the involvement of the left hippocampus and splenium of the corpus callosum. The lesion demonstrates an intrinsic T1 hyperintense signal on T1-weighted imaging and a susceptibility artifact on Swan, indicating the presence of blood products. B. PET CT with FDG tracer uptake acquired 102 and 253 minutes after the injection demonstrates prominent focal uptake surrounding the region of the calcified tumor. C. Follow-up imaging confirms disease progression with interval increase in the size of the tumor and the surrounding enhancement. FLAIR: fluid attenuated inversion recovery; FDG: fludeoxyglucose; PET: positron emission tomography

Patient B was diagnosed with localized pineoblastoma with the DICER1 somatic mutation at 20 months of age, after presenting with two weeks of progressive weakness. He underwent gross total resection of his large posterior fossa mass and had a postoperative course complicated by intraventricular hemorrhage (Figure 3). He subsequently received chemotherapy as per ACNS0334. MRI after three cycles of induction chemotherapy showed evidence of subtle progression in the tumor bed apparent on the diffusion sequence. Given the subtle nature of finding, the decision was made to continue with high-dose chemotherapy with autologous stem cell rescue. Spine MRI at this time showed no disseminated disease. The patient underwent conditioning with carboplatin and thiotepa prior to stem cell infusion. His chemotherapy plan was interrupted due to persistent adenoviremia and he, therefore, underwent focal radiation therapy (5400 cGy) to the surgical bed while undergoing treatment for the infection. He received his second of three planned “tandem transplants” after the completion of radiation therapy but not the third due to adenoviral contamination. Routine MRI obtained three months following the completion of therapy showed concern for disease progression versus treatment effect. Several short-interval, follow-up scans continued to demonstrate vague areas concerning for disease progression, though it was radiographically difficult to differentiate from treatment effect. Delayed PET imaging was ultimately obtained, which showed FDG uptake surrounding the calcified mass of the left occipital region, with uptake increasing between early and delayed imaging, suspicious for recurrent tumors (Figure 4). Using the formula published in Higashi et al., the SUV retention index was calculated at 2.1 (SUV early=5.972, SUV late=6.098) [7]. This increase in SUV over time aligns with what has been published in the literature in prior cases of malignant lesions who have undergone delayed FDG PET imaging. The patient was treated with three cycles of oral etoposide until he was noted to have further progression with leptomeningeal dissemination on MRI seven months later. He received one cycle of oral topotecan with continued and significant tumor progression, ultimately leading to his death almost two years after his diagnosis.

## Discussion

Delayed FDG PET imaging

Kubota et al. evaluated whole-body FDG PET images at one hour and two hours after injection in 22 subjects. They found that most malignant lesions, including metastatic mediastinal lymph nodes, lymphomas, and lung cancer lesions, had higher FDG uptake at two hours as compared with one hour post-injection. They found the opposite to be true for benign lesions, with the exception of sarcoidosis, as well as normal tissues. With the increase in the tumor to background contrast from one to two hours post-injection, the sensitivity for tumor detection increased significantly. All malignant lesions became clearer, and three lesions that were called equivocal on one hour post-injection images were clearly detected two hours post-injection [8]. Basu et al. prospectively investigated FDG uptake in tumors and normal organs over an eight-hour period in patients with non-small cell lung carcinoma (NSCLC). The results showed that tumor sites had increased uptake of FDG over this eight-hour time period, compared to surrounding normal tissues that demonstrated decreasing or stable FDG values. This increase in the contrast between the lesion and background could potentially lead to improved test sensitivity for detecting malignant lesions [9]. Suga et al. aimed to clarify the difference in F-18 FDG uptake kinetics between FDG-avid NSCLC and benign lesions and to determine the optimal parameter for differentiation. They found that a delayed PET/CT scan enhances the difference of FDG uptake between malignant and benign lesions, with the optimal parameter for differentiation being delayed SUVmax>5.5, though they did find several benign lesions were indistinguishable from NSCLC [10]. Parghane et al. summarized the false-positive and false-negative results of standard FDG PET/CT in characterizing musculoskeletal lesions and considered the values and limitations of dual-time-point (DTP) FDG PET for differentiating malignant from benign musculoskeletal lesions. They reiterated that increased sensitivity for lesion detection is a benefit of delayed-time point imaging and thoughtfully considered the potential errors in the interpretation of results. They found the causes for false positives to be inflammatory or infectious lesions as well as locally aggressive benign tumors and noted low-grade sarcomas as the cause of false negatives. They did note that delayed imaging could be helpful when needing to differentiate malignancy from sites of chronic inflammation and post-treatment residual disease versus necrosis [11].

Delayed FDG PET in pediatric patients

Constantini et al. prospectively compared dual-time-point FDG PET/CT in pediatric patients with benign and malignant entities (not including brain tumors). The retention index was calculated by dividing the difference between delayed and early SUV, dividing by early SUV, and multiplying by 100. A retention index of 10% was chosen to delineate the benign and malignant processes. They noted an increase in average SUV between two time points in patients with malignant disease, compared with an overall stable average SUV for patients with benign lesions. The retention index was calculated by dividing the difference between delayed and early SUV, dividing by early SUV, and multiplying by 100. A retention index of 10% was chosen to delineate benign and malignant processes. The average retention index was 37.1% ± 10.8% for patients with malignant lesions versus −9.9% ± 7.1% for benign lesions and concluded that dual-time-point FDG PET/CT is useful in differentiating benign and malignant pediatric processes [12]. Shulkin and colleagues compared standard and delayed FDG PET/CT in pediatric osteosarcoma patients. They compared serial FDG PET/CT imaging in patients with newly diagnosed osteosarcoma, obtained at diagnosis and then at five and 10 weeks after the start of therapy. They found that FDG uptake did not significantly vary between standard and delayed imaging timepoints during any of these scans [13]. In each of these cases, the patients did not require anesthesia to undergo imaging.

Delayed FDG PET imaging in adult CNS tumors

The use of FDG PET imaging in brain tumors is controversial due to the high brain uptake, which can obscure potentially pathologic findings. However, FDG PET/CT is widely used in all areas of oncology and as a readily available resource, it continues to be discussed and considered a potential clinically pertinent imaging modality. Kim et al. sought to explore the usefulness of using dual-time-point FDG positron emission tomography imaging (DTPI) to grade brain tumors. They prospectively looked at 22 lesions of 18 consecutive patients with primary or metastatic brain tumors. They found that the SUVs of the delayed images were more efficient than those of early images to classify lesions by tumor grade, therefore concluding that DTPI may be a better imaging method to grade the brain tumor than early imaging only [14]. Spence et al. hypothesized that the delineation of gliomas from gray matter with FDG PET could be improved by extending the interval between FDG administration and PET data acquisition. Their visual analysis of 19 adult patients with supratentorial gliomas showed that the delayed images better distinguished the high uptake in tumors relative to uptake in gray matter, and their SUV comparisons showed a greater uptake in the tumors than in gray matter, brain, or white matter at the delayed times. They concluded that tumor enhancement is greater than the enhancement of surrounding brain regions at later imaging times, consistent with a greater effect of FDG-6-phosphate degradation on normal brain relative to glioma [15]. Malkowski et al. used single- and dual-time-point acquisition of 18F-FET PET parameters to differentiate between primary low-grade gliomas (LGGs) and high-grade gliomas (HGGs). The PET examination was deemed positive for glioma if the metabolic activity was 1.6 times higher than that of the background (contralateral) brain. The maximum tissue to brain ratios (TBRmax) were calculated at both 10 and 60 minutes after the administration of the isotope. They found the overall sensitivity of PET was 97% and that several analyzed parameters were significantly different between LGGs and HGGs, thus concluding that 18F-FET PET is valuable for the non-invasive determination of glioma grade, especially when dual time-point metrics are used [16].

Barker and colleagues attempted to utilize standard timepoint FDG PET imaging to distinguish tumor progression from radiation injury following initial radiation therapy for malignant glioma. FDG PET scans were obtained in 55 patients with malignant glioma for whom MRI obtained after initial surgery and radiation therapy demonstrated enlarging, enhancing lesions consistent with either tumor progression or radiation necrosis. In univariate analysis, the FDG PET score was a significant predictor of survival time after FDG PET scanning (P=0.005). Median survival was 10 months for patients with FDG PET scores of 2 or 3 (glucose uptake>/=adjacent cortex) and 20 months for those with scores of 0 or 1 (glucose uptake</=adjacent cortex). They concluded that FDG PET scanning has prognostic value in a cohort limited to patients with suspected recurrent high-grade glioma [17]. Horky et al. studied the ability of dual phase FDG PET/CT imaging to accurately distinguish tumors versus necrosis in patients treated for brain metastases. They included 32 consecutive patients with treated brain metastases, lesion size greater than 0.5 cm, and a suspected recurrence on MRI underwent dual-phase FDG PET/CT. They found that early or late SUVs of the lesion alone did not differentiate between tumor and necrosis. They did note that regardless of histological type, differentiation of necrosis from metastatic brain lesions was improved by using the change of lesion to gray matter SUVmax ratios as a function of time [18]. Ricci et al. also sought to evaluate the ability of FDG PET to differentiate recurrent tumors from radiation necrosis. In 84 patients with a history of a treated intracranial neoplasm in whom recurrent tumor or radiation necrosis was suggested by clinical or MRI findings, they looked at PET imaging and qualitatively compared the metabolic activity of the PET abnormality with normal contralateral gray and white matter. PET findings were confirmed histologically in 31 patients. They found that about 30% of the patients would have been received inappropriate medical treatment had the PET scan been the sole determinant of therapy. Their data suggested the limited ability of standard FDG PET to differentiate recurrent tumors from radiation necrosis, with high false-positive and false-negative PET scan results [19].

Bochev et al. evaluated 38 patients with brain neoplasms and non-tumor structural lesions who underwent a selective brain FDG PET/CT at two time points (60 and 180 minutes) after administration. They found that a visual analysis showed a better delineation of malignant lesions on late registrations with a higher inter/intraobserver agreement as compared to the early images. A semiquantitative analysis demonstrated significant differences in early and late indices of metastases and gliomas but failed in distinguishing gliomas from metastatic lesions and benign lesions [20].

## Conclusions

Delayed FDG PET imaging has been helpful in determining radiation effect versus disease recurrence, and we presented two cases where a delay in imaging has helped with an earlier and simpler diagnosis of relapsed disease. The noninvasive nature and omnipresence of FDG PET imaging are positive aspects, though limitations do exist. Not all studies have found striking differences in the FDG PET images of benign and malignant processes, and there is a variation between oncologic processes. Another limitation to consider is that with pediatric patients who require anesthesia, increasing the time under general anesthesia to complete this study carries its own risks. There are cases, however, where short of biopsies of lesions, it is extremely difficult to label recurrence versus treatment effect. This often leads to the necessity for short-term interval follow-up scans and creates a significant amount of anxiety for patients and their families. A delay in diagnosis can also lead to more widespread disease once relapse is called. In summary, delayed FDG PET imaging should be considered in complicated patients, though the results should be interpreted with caution.

## References

[1] Langleben DD, Segall GM (2000). PET in differentiation of recurrent brain tumor from radiation injury. J Nucl Med.

[2] Verma N, Cowperthwaite MC, Burnett MG, Markey MK (2013). Differentiating tumor recurrence from treatment necrosis: a review of neuro-oncologic imaging strategies. Neuro Oncol.

[3] Chao ST, Suh JH, Raja S, Lee SY, Barnett G (2001). The sensitivity and specificity of FDG PET in distinguishing recurrent brain tumor from radionecrosis in patients treated with stereotactic radiosurgery. Int J Cancer.

[4] Zhuang H, Yuan X, Chang JY (2016). Exploration of the recurrence in radiation brain necrosis after bevacizumab discontinuation. Oncotarget.

[5] Kumar Y, Gupta N, Mangla M, Hooda K, Mangla R (2017). Comparison between MR Perfusion and 18F-FDG PET in differentiating tumor recurrence from nonneoplastic contrast-enhancing Tissue. Asian Pac J Cancer Prev.

[6] Schillaci O (2012). Use of dual-point fluorodeoxyglucose imaging to enhance sensitivity and specificity. Semin Nucl Med.

[7] Higashi T, Saga T, Nakamoto Y (2002). Relationship between retention index in dual-phase 18F-FDG PET, and hexokinase-II and glucose transporter-1 expression in pancreatic cancer. J Nucl Med.

[8] Kubota K, Itoh M, Ozaki K (2001). Advantage of delayed whole-body FDG-PET imaging for tumour detection. Eur J Nucl Med.

[9] Basu S, Kung J, Houseni M, Zhuang H, Tidmarsh GF, Alavi A (2009). Temporal profile of fluorodeoxyglucose uptake in malignant lesions and normal organs over extended time periods in patients with lung carcinoma: implications for its utilization in assessing malignant lesions. Q J Nucl Med Mol Imaging.

[10] Suga K, Kawakami Y, Hiyama A (2009). Dual-time point 18F-FDG PET/CT scan for differentiation between 18F-FDG-avid non-small cell lung cancer and benign lesions. Ann Nucl Med.

[11] Parghane RV, Basu S (2017). Dual-time point 18F-FDG-PET and PET/CT for differentiating benign from malignant musculoskeletal lesions: opportunities and limitations. Semin Nucl Med.

[12] Costantini DL, Vali R, Chan J, McQuattie S, Charron M (2013). Dual-time-point FDG PET/CT for the evaluation of pediatric tumors. Am J Roentgenology.

[13] Shulkin B, Davis J, Navid F, Billups C, Santana V, Reddick G, Daw N (2013). FDG PET/CT in pediatric osteosarcoma: Comparison of standard and delayed scanning. J Nucl Med.

[14] Kim DW, Jung SA, Kim CG, Park SA (2010). The efficacy of dual time point F-18 FDG PET imaging for grading of brain tumors. Clin Nucl Med.

[15] Spence AM, Muzi M, Mankoff DA (2004). 18F-FDG PET of gliomas at delayed intervals: improved distinction between tumor and normal gray matter. J Nucl Med.

[16] Malkowski B, Harat M, Zyromska A, Wisniewski T, Harat A, Lopatto R, Furtak J (2015). The sum of tumour-to-brain ratios improves the accuracy of diagnosing gliomas using 18F-FET PET. PLoS One.

[17] Barker FG 2nd, Chang SM, Valk PE, Pounds TR, Prados MD (1997). 18-Fluorodeoxyglucose uptake and survival of patients with suspected recurrent malignant glioma. Cancer.

[18] Horky LL, Hsiao EM, Weiss SE, Drappatz J, Gerbaudo VH (2011). Dual phase FDG-PET imaging of brain metastases provides superior assessment of recurrence versus post-treatment necrosis. J Neurooncol.

[19] Ricci PE, Karis JP, Heiserman JE, Fram EK, Bice AN, Drayer BP (1998). Differentiating recurrent tumor from radiation necrosis: time for re-evaluation of positron emission tomography?. AJNR Am J Neuroradiol.

[20] Bochev PH, Klisarova A, Kaprelyan AG, Chaushev B, Dancheva Z (2013). Delayed FDG-PET/CT images in patients with brain tumors - impact on visual and semiquantitative assessment. J of Imab.

